# How Are We Educating Our Children?1Read before the Section on Medicine, Buffalo Academy of Medicine, April 9, 1901.

**Published:** 1901-07

**Authors:** A. W. Bayliss

**Affiliations:** Buffalo, N. Y.


### HOW ARE WE EDUCATING OUR CHILDREN?[1]

BY A. W. BAYLISS, M. D., Buffalo, N. Y.

ONE of the most important duties of our life is the proper education of the young. To educate them properly not only mentally but physically should be our aim. It has often occurred to me within the past ten years that we are too one-sided in our work in that line. In the days of our forefathers the education they received was such as better fitted them for the battle of life that was before them; that is, they were more able to stand the rough and almost turbulent life into which they were to enter than are we of today. Perhaps you may think me something of a pessimist, but I trust I am not; I think there are few who take a brighter view of life than I do. If we were all to let affairs rest as they are when we think they may be improved, without adding our mite to change them, I fear we would wear the ruts pretty deep, so deep it would be hard to leave them.

Since the formation of the world there have been the different ages and this seems to be the age of education; but it appears to extend only in one direction, which I think is a mistake. I would not have the young of today necessarily athletes, yet if they are and turn toward innocent athletic exercises, I think that is one way of developing manhood and I was going to add womanhood, but that is carrying it almost too far, so we will stop at girlhood, the proper teaching of which should most certainly be in our public schools. It should not be optional but obligatory upon all, unless for certain reasons a scholar may be excused. How often are we called to attend the child who, as one teacher very tactfully put it, "does not study too much but exercises too little," one who is on the verge of breaking down under the strain to which they are subjected? It may be in one form or it may be in another, but easily traced to the cause which is debilitating our young. When the child has spent several hours in study he feels but little like exercise, no matter how much good it may do him to stir about in the fresh air, of which we get but little in a city of this size. If he can find the couch or bed he feels more like occupying it than taking the necessary exercise.

1. Read before the Section on Medicine, Buffalo Academy of Medicine, April 9, 1901.

How seldom if ever are we called to attend the child in the lower walks of life for this disease. I don't mean the middle-class, but the lower, those who must help to earn the living that they have. Some may say these children are dull and not capable of being wrought up to that stage of excitement, if you please, that their more lucky neighbors are, yet their teachers all speak well of them as to their brightness and capability of learning, and how many of them do we see that become prosperous business men? No, the reason is they are not like the fruit tree grown in the forest, whose branches may reach far enough above its more humble brother in the orchard to overlook a vast expanse of territory, yet of what use is it, it being so exposed in its weakened form it cannot withstand the storms of life as well as its brother, and often the fruit that it should bear blasts for want of nature's most necessary element, strength. And if fruit does form upon this weakling, it is apt to be like the parent from which it came, tender and liable to be ruined by the storms which it certainly must encounter. I would not take from the present hours of study of our schools, but rather add to, by, as I said before, adding to each school a systematic course in physical development; not a brief ten to fifteen minutes, but a sufficient time to develop the physical system to the same level that the mental is. Some may say they have not the time, but who of you that cannot do more mental work in one hour when you are fresh and your mind clear than you can do in three or four times that length of time when tired. Therefore I would divide the time differently, taking from the hours of study enough to give a sufficient time for exercise, and have properly qualified teachers to teach the subject. I am told that a certain amount of exercise in the schools is compulsory, but the time is too short and those who teach it are not qualified to do so. I speak of the majority of teachers.

Dr. E. A. Edline, before the Illinois State Medical Society, read a paper last May upon Physical Culture in which he considers it necessary for the young, as the present state of affairs tends greatly to weaken the race, to which I think you will all agree. Also Mr. Beitzel, Principal of the Mechanicsburg school, read a paper before the associated health authorities, in which he recommends medical inspection and considers that we are developing the mental powers at the expense of the physical system. In several cities they have physicians appointed to inspect the schools, but their work is confined to the prevention of contagious diseases, examination of the sight and hearing, also sanitation.

In some of the German cities, they have the same inspection, but it is, generally speaking, in an experimental stage, being a success so far as they have gone with it. But I fail to find anywhere that they have anything in the line of inspection in regard to the physical needs of the child. Why would it not be well to have inspectors appointed who are qualified to say what form of exercise a child should take? Perhaps instructors in physical culture would be qualified, but I fear they would be inclined to overtax the child in their enthusiasm to accomplish much; and, again, they would not understand so well the condition of the child's system; therefore, I would advise the appointment of physicians who are specially qualified for the work. In nearly all cities where they have inspectors at work there is a certain amount of clashing between the health authorities and the school department. I think perhaps the reason of this is the school department fails so often to see the necessity of suspending the child from school; but this in time will be overcome and I think the two departments will work in unison.

As the wheels of all public machinery—especially these departments—move slowly, I think it quite necessary that some action be taken in this matter, and I trust a discussion of this subject will be entered into this evening. I have purposely made my paper short that all may express their opinion upon this, I think, vital question. I, as you see, have omitted to speak of the sanitary condition of the schools, for the reason that the question is being agitated now, and in due time will be presented to the public.

### HOW TO STUDY.

After speaking with several teachers, I find they all say they do not crowd their pupils but rather hold them back; but any fair, liberal-minded person I think will see that this is not so. From the time the child enters the kindergarten to the time he leaves the high school, it is a continuous process of craming or developing grey matter. The same brain cells are not brought into use in the study of one subject that are in the study of a different one, yet the child is at all times under the same mental strain when at work. I spoke of the child in the kindergarten as being hurried through life. If you doubt that, look at the little tots as they come from school with tears in their eyes, when they have been told that if they did not do better they would have to go back into a lower class or remain in the class they are in while their more fortunate companions are promoted, and tell me then that they are held back! This is a picture that may be seen quite often these days. This same process of teaching is seen throughout school life only in different degrees, as the teacher is more or less ambitious for her well-being and situation, also her future advancement, for we are measured by our failures as well as by our victories; so it behooves them to do their best if they expect to prosper in their chosen field of labor.

Instead of this process of grinding, I would have more time spent in teaching them how to study. Not so much to work, but how to work. Of course, the present text-books are much plainer to the child than were the old ones. Compare the old Brown's grammar from which the beginner graduated in time with the present text-books on language. Now the pupil goes step by step to the more advanced subject. Yet the technical way to study is not taught as it should be. How often do we see the girl or boy sitting listlessly over their books with thoughts far away, yet call this study, when if they knew how to work, for work it is, how much less time would be spent over their books than now. What would we think today of putting a teacher into a school, no matter how learned he or she may be if they were not taught how to teach, yet we expect the child to grasp the lesson in his undeveloped state. Therefore let us teach the child first how to study and then when to study.

The present system of marking the standing of pupils should be abolished, also all honor rolls, and instead a private record kept in each school or room for reference, and when one falls below a certain per cent., the parents should be notified and requested to spur up the careless child, if careless he is, instead of making it public before the whole school and neighborhood and holding the child up to the ridicule of his mates, which must act to discourage many, for who of us can stand a continued faultfinding without becoming discouraged? And how much more vim and life it puts into us if we are led to think we sometimes do something worth commending. If it acts so on us it certainly must do the same with the child, for the only difference between us is we are a little larger than they and have seen a little more of life. Then the child is not so much educated by this system that we have in use now. Up to the time the child commences to take the regents' examinations there is more of a general education; but when that begins, the child's attention is directed more especially upon the study in which he expects to take an examination, and there is a continuous drill upon that subject until they pass it, and what can be worse for the child than this form of study? It does not teach the principle only so far as it is necessary to pass. Not only the sin of omission is seen here but the sin of commission also, for you will all allow, I think, that a child kept under this continuous strain must be debilitated and weakened, when if this were different the child would have less to worry about as well as be better prepared for that which is to come.

For my part I cannot see what need we have of the board of regents in a city of this size. They issue certificates which are an educational legal tender in this state, but of no use elsewhere. A child spends twelve to fourteen years for one of these certificates, and then is told if he wishes to enter a college outside of the state that he must pass an examination. How would you like to work the same time for a few dollars that you must spend in this state, resembling the old bank notes in use years ago, when if you entered another state you must have your money changed to that which was legal tender there? No. If we cannot have a legal tender for our entire country, I would ask to be as a city released from the supervision of the regents' board, and set the standard high, so high by doing away with the monthly, and if possible the term examinations, and having a final one; that when our boys and girls go to another state to enter a college or university, they would have the pure standard metal that would shine not only at home but wherever they may be.

592 Spring Street.

